# Biopsy-proven Henoch-Schönlein purpura nephritis: a single center experience

**DOI:** 10.1007/s00467-020-04809-8

**Published:** 2020-10-21

**Authors:** Eda Didem Kurt-Şükür, Thivya Sekar, Kjell Tullus

**Affiliations:** 1 Department of Pediatric Nephrology, Dr. Sami Ulus Children’s Hospital, Ankara, Turkey; 2 Department of Pediatric Nephrology, Great Ormond Street Hospital for Children NHS Foundation Trust, London, UK; 3 Department of Pediatric Pathology, Great Ormond Street Hospital for Children NHS Foundation Trust, London, UK

**Keywords:** Immunoglobulin A vasculitis, HSPN, Chronic kidney disease, Prognosis, Treatment, Outcome, Pediatrics

## Abstract

**Background:**

Knowledge on normal progress and treatment of Henoch-Schönlein purpura nephritis (HSPN) is limited. This study reviews outcome, clinical, pathological, and therapeutic factors affecting the prognosis of HSPN patients.

**Methods:**

Forty-nine children with biopsy-confirmed HSPN diagnosed between September 2008 and 2018 were included. Demographics, clinical and laboratory data, treatment, and outcome were recorded at the time of biopsy, 3, 6, 12, and 24 months and at last visit. Clinical outcome was graded according to Meadow’s criteria.

**Results:**

The median age at time of biopsy was 10.1 years (IQR:5.7) and female/male ratio 24/25. At presentation, 40.8% of patients had nonnephrotic proteinuria, 18.4% nephrotic syndrome (NS), 4.1% nephritic syndrome (NephrS), and 36.7% NephrS+NS. There were 11 patients with an estimated glomerular filtration rate below 90 ml/min/1.73 m^2^. Biopsy specimens were classified according to International Study of Kidney Diseases in Children (ISKDC) and Oxford Classification MEST-C scoring systems. Forty-one patients received angiotensin-converting enzyme inhibitors/angiotensin receptor blockers, 37 patients steroids, and 35 patients other immunosuppressive medications. At last visit, 24 patients had stage 1 chronic kidney disease (CKD), three stage 2 CKD, and two had stage 5 CKD. Neither clinical parameters nor ISKDC biopsy grade or treatment modalities effected the final outcome. The Oxford classification showed significantly increased segmental glomerulosclerosis in patients with unfavorable outcome. Favorable outcome was associated with shorter time from kidney involvement to biopsy and start of treatment.

**Conclusion:**

A large proportion of patients continued to show signs of CKD at last follow-up while only a small proportion developed stage 5 CKD.

## Introduction

Henoch-Schönlein Purpura (HSP) or IgA vasculitis is the most common vasculitis seen in children, with an estimated annual incidence of 3 to 26.7 per 100.000 children [1]. The disease is diagnosed in a child with palpable purpura (mandatory criterion) and at least one of the following criteria; diffuse abdominal pain, any biopsy showing predominant IgA deposits, arthritis/arthralgia (acute, any joint) and kidney involvement (hematuria ± proteinuria) [2]. In doubtful cases with an atypical distribution of the skin rash, a biopsy sample showing Ig A deposition becomes a required criterion [3].

Kidney involvement is the most serious complication and its occurrence varies from 20 to 50% [3–7]. A majority of children have a mild renal presentation and a very good chance for recovery [7, 8]. The first urinary findings are seen within 4 weeks of disease onset in the majority of patients, and 97% of cases have presented within 6 months [9]. Progression to chronic kidney disease (CKD) has, in different case series, been reported in 5 to 45% of children with any degree of HSP nephritis (HSPN) [9–13]. The risk of poor kidney outcome has been reported to be highest in children with nephritic/nephrotic presentation and lowest in those with only microscopic hematuria ± minimal proteinuria [11, 12].

Treatment of HSPN is not well defined as randomized controlled trials are scarce. Pediatric nephrologists face a dilemma of delaying treatment in a small group of children with active inflammatory disease which could increase their chance of developing CKD. This needs to be balanced against a high chance of overtreatment of children with spontaneous regression of their nephritis. The lack of proven effective treatment together with substantial potential side-effects from the treatments used makes the decision even more difficult.

The aim of this study is to review the outcome of biopsy-proven HSPN children seen at Great Ormond Street Hospital for Children to describe the chances of developing CKD in the short- to medium-term perspective and to see if we could find any signal that the treatments used had made any difference.

## Methods

Children with a biopsy-proven diagnosis of HSPN between September 2008 and September 2018 were included in the study. The study was registered with the hospital’s Clinical Audit Committee (audit ref. no: 2502). Patients with an age of < 18 years at time of disease onset were included. A follow-up time of more than 6 months was required for inclusion. Demographic characteristics, clinical and laboratory data, treatment, and outcome were recorded from the hospital records at the time of biopsy, at 3, 6, 12, and 24 months follow-up and at last visit.

The validated EULAR/PRINTO/PRES criteria for HSP diagnosis were used [3]. Kidney involvement was graded as isolated hematuria, mild hematuria ± proteinuria, nephritic syndrome (NephrS), nephritic syndrome + nephrotic range proteinuria (spot urine protein/creatinine > 200 mg/mmol or albumine/creatinine > 100 mg/mmol), nephrotic syndrome (NS), and kidney failure by Kidney Disease: Improving Global Outcomes (KDIGO) criteria [14, 15]. Hematuria was defined when microscopic examination showed more than 5 red blood cells (RBC)/ul in a fresh uncentrifuged urine sample or positivity on dipstick [16]. Proteinuria was defined by positivity on dipstick method or > 3 mg/mmol albumin/creatinine in spot urine [17]. Nephritic syndrome was diagnosed by hematuria, accompanied by hypertension and/or edema, oliguria, and varying degrees of abnormal kidney function. Nephrotic syndrome diagnosis was made when there was edema, hypoalbuminemia (< 25 g/L), and heavy proteinuria (spot urine protein/creatinine > 200 mg/mmol) [14]. Hypertension (HT) was diagnosed according to the latest AAP guidelines; normal blood pressure (BP) as < 90th percentile for children 1–13 years and < 120/80 mmHg for children older than 13 years [18]. Estimated glomerular filtration rate (GFR) was calculated using the modified Haycock-Schwartz formula [19]. Acute kidney injury was defined by modified RIFLE criteria (Risk, Injury, Failure, Loss of kidney function, End-Stage Kidney disease) [20]. The diagnosis of CKD was made when there was abnormal kidney function lasting for more than 3 months and was graded as follows; stage 1—kidney damage with normal or increased GFR (≥ 90 ml/min/1.73 m^2^), stage 2—mildly decreased GFR (60–89 ml/min/1.73 m^2^), stage 3a—mildly moderately decreased GFR (45–59 ml/min/1.73 m^2^), stage 3b—moderately severely decreased GFR (30–44 ml/min/1.73 m^2^), stage 4—severely decreased GFR (15–29 ml/min/1.73m^2^), and stage 5—kidney failure (< 15 ml/min/1.73 m^2^) [21]. Kidney replacement modalities, peritoneal/hemodialysis, or kidney transplantation were noted.

Kidney biopsy indications were NephrS, NS, persistent severe proteinuria (urine protein/creatinine > 200 mg/mmol for 4 weeks), persistent moderate proteinuria (urine protein/creatinine 100–200 mg/mmol for 3 months), and acute kidney injury by KDIGO [15]. Pathological evaluation of kidney biopsies was made by a pediatric pathologist (TS) according to International Study of Kidney Diseases in Children (ISKDC) and the Oxford Classification [22, 23]. The classification according to ISKDC was grade 1 (minimal histological alterations), grade 2 (mesangial proliferation without crescents), grade 3 (mesangial proliferation with < 50% crescents), grade 4 (mesangial proliferation with 50–75% crescents), grade 5 (mesangial proliferation with > 75% crescents), and grade 6 (membranoproliferative-like glomerulonephritis). The Oxford classification gives a MEST score from grading mesangial hypercellularity score (M: M0 < 50% of glomeruli, M1 > 50% of glomeruli), endocapillary proliferation (E: E0: absent, E1: present), segmental glomerulosclerosis defined as adhesion or sclerosis (S: S0: absent, S1: present), extent of tubular atrophy/interstitial fibrosis (T: T0 < 25%, T1: 25–50%, T2 > 50%) and crescents (C: C0: absent, C1: crescents in < 1/4 of glomeruli, C2: > 1/4 of glomeruli) [23]. Importantly the Oxford classification includes grading of chronic changes.

Clinical outcome was graded according to Meadow’s criteria; A: normal (no clinical or laboratory abnormality), B: minor urinary abnormalities (urine albumin/creatinine: 3–30 mg/mmol ± hematuria), C: active kidney disease (urine albumin/creatinine > 30 mg/mmol, hypertension or elevated plasma creatinine with eGFR ≥ 60 ml/min/1.73 m^2^), D: abnormal kidney function (eGFR < 60 ml/min/1.73 m^2^) [24]. Complete remission and favorable outcome was defined as grade A, whereas grades B–D were considered as no remission, unfavorable outcome.

### Statistical analyses

Data analyses were performed by using SPSS Version 21.0 (IBM Corporation, Armonk, NYC, USA). Samples were tested with Shapiro–Wilk test to determine normality of distributions. According to the results, nonparametric tests were preferred. Continuous variables were compared by Mann–Whitney *U* test and categorical variables by chi-square or Fisher’s exact test as appropriate. A *P* value of < 0.05 was considered statistically significant.

## Results

Forty-nine children with biopsy-proven HSPN were followed for at least 6 months, median follow-up period was 22 (IQR:27) months. Table 1 shows the demographic characteristics, laboratory results, and treatments given. Median age at biopsy was 10.1 (IQR:5.7) years. Fourteen patients (28.6%) showed recurrent attacks of purpura. Twenty-five children had arthritis, 23 gastrointestinal (GI) symptoms (four GI bleeding), and four scrotal involvement. Among all, 23 patients (46.9%) had kidney involvement at the time of HSP diagnosis. Clinical presentation of HSPN was proteinuria in 20 (40.8%), NS in 9 (18.4%), NephrS in 2 (4.1%), and NephrS+NS in 18 (36.7%) patients. At the time of biopsy 16.3% of the patients had stage 1, 8.2% stage 2 hypertension, and the rest were normotensive, 22.4% of children had an eGFR < 90 ml/min/1.73 m^2^ and 63% low serum albumin levels (< 35 g/L). All C-reactive protein (CRP) values were normal, and 83% of erythrocyte sedimentation rate (ESR) values were less than 10 mm/h. The white cell counts were above 10,000/ mm^3^ in 44.6% of the children.

**Table 1:** Demographic and laboratory characteristics and treatment modalities of the patients (at the time of biopsy)

	Patients, *n* = 49
Gender, female male	24 (49%) 25 (51%)
Age, years	10.1 (5.7)
Ethnicity	
European descent	30 (61.25%)
Other ethnic groups	6 (12.25%)
Unknown (not asked/ not specified)	13 (26.5%)
Duration from HSPN to biopsy, months	1 (5)
Duration from HSP to HSPN, months	1 (1)
Systolic blood pressure, mm Hg	108 (19)
Serum creatinine, umol/L	61 (32)
eGFR, ml/min/1.73 m^2^	142 (71.5)
Serum albumin, g/L	30 (11.75)
White blood cell count, n/uL	10,800 (11250)
Serum C-reactive protein, mg/L	5.5 (13.75)
Eryhtrocyte sedimentation rate, mm/h	38.5 (64.75)
Urine albumin/creatinine, mg/mmol	367 (594)
Biopsy grade	
1	1(2%)
2	7(14.3%)
3	32(65.3%)
4	5(10.2%)
5	4(8.2%)
Treatment	
ACE-I/ARBs	41 (83.7%)
Oral steroids	20 (40.8%)
Pulse+oral steroids	17 (34.7%)
No steroids	12 (24.5%)
Other immunosuppresive agents	35 (71.4%)
Mycophenolate mofetil	20 (40.8%)
Cyclophosphamide	6 (12.2%)
Azathioprine	3 (6.1%)
Tacrolimus	3 (6.1%)
Rituximab	2 (4.1%)
Cyclosporine	1 (%)
Omega 3	9 (18.4%)
Plasma exchange	5(10.2%)
Dialysis	1 (2%)
Transplant	1 (2%)

*ACE-I*, Angiotensin converting enzyme inhibitor; *ARB*, angiotensin receptor blocker; *eGFR*, estimated glomerular filtration rate; *HSPN*, Henoch-Schönlein Purpura Nephritis. Continuous data are presented as median (IQR: interquartile range); categorical data are given as frequency (percentage)

Table 2 depicts the patient characteristics according to severity of histopathological grading. According to the ISKDC classification 8 children were classified as having a mild (grades 1–2) disease according to kidney pathology while 41 showed biopsy findings of grades 3–5. At the time of biopsy, the eGFR and serum albumin levels in the group with mild pathological findings were significantly higher than in children with severe biopsy findings. Serum albumin, creatinine, and eGFR levels were within normal limits in all children with mild pathology, whereas in the severe pathology group 48.7% had hypoalbuminemia and 23% eGFR below 90 ml/min/1.73 m^2^. There were four children with grade 5 pathology, all of whom presented with NS-NephrS, three with eGFR less than 90 ml/min/1.73 m^2^. The Oxford classification defined M1 in 45 (91.8%), E1 in 28 (57.1%), S1 in 17 (34.6%), C0 in 8 (16.3%), C1 in 24 (49%), and C2 in 17 (34.%) of the biopsies. None of the patients showed any signs of tubular atrophy, T0 was thus seen in all of the pathology specimens.

**Table 2:** Characteristics of patients according to histopathological staging according to International Study of Kidney Diseases in Children (ISKDC)

	Mild HSPN (grade 1–2) *n* = 8	Severe HSPN (grade 3–5) *n* = 41	*P* value
Gender, female	6 (75%)	18 (43.9%)	0.108
male	2 (25%)	23 (56.1%)	
Age at biopsy, years	10.8 (6.1)	8.6 (5.8)	0.091
eGFR at biopsy, ml/min/1.73 m^2^	162.5 (39.5)	134 (74.5)	0.030
Duration from HSP to HSPN, months	0 (22)	1 (1)	0.989
Duration from HSPN to biopsy, months	11.5 (34.7)	1 (3.5)	0.009
Urine albumin/creatinine at biopsy, mg/mmol	127 (141)	402 (663)	<0.001
Serum albumin at biopsy, g/L	42 (10)	30 (9.5)	<0.001
Follow-up, months	18.5 (15)	24 (29)	0.267
Clinical presentation			<0.001
Mild proteinuria	3 (37.5%)	0 (0%)	
Nephrotic range proteinuria	4 (50%)	7 (17.1%)	
Nephrotic syndrome	0 (0%)	9 (22%)	
Nephritic syndrome	1 (12.5%)	1 (2.4%)	
Nephritic syndrome+nephrotic range proteinuria	0 (0%)	6 (14.6%)	
Nephritic+nephrotic syndrome	0 (0%)	18 (43.9%)	
Treatment(s)			
ACE-I/ARBs	8 (100%)	33 (80.5%)	0.172
Oral steroids	4 (50%)	16 (39%)	0.563
Pulse+oral steroids	0 (0%)	17 (41.5%)	0.038
No steroids	4 (50%)	8 (19.5%)	0.067
Mycophenolate mofetil	0 (0%)	20 (48.8%)	0.015
Azathioprine	0 (0%)	3 (7.3%)	0.430
Cyclophosphamide	0 (0%)	6 (14.6%)	0.248
Cyclosporine	0 (0%)	1 (2.4%)	0.655
Tacrolimus	0 (0%)	3 (7.3%)	0.430
Rituximab	0 (0%)	2 (4.9%)	0.524
Omega	4 (50%)	5 (12.2%)	0.028
Meadow’s score			0.410
A	2 (25%)	18 (43.9%)	
B	6 (75%)	18 (43.9%)	
C	0 (0%)	3 (7.3%)	
D	0 (0%)	2 (4.9%)	

*ACE-I*, Angiotensin converting enzyme inhibitor; *ARB*, angiotensin receptor blocker; *eGFR*, estimated glomerular filtration rate; *HSPN*, Henoch-Schönlein Purpura Nephritis. Continuous data are presented as median (IQR: interquartile range); categorical data are given as frequency (percentage). Meadow’s grade A: normal, B: minor urinary abnormalities, C: active kidney disease, D: kidney insufficiciency

Eight children received treatment with more than four different drugs and, 16 had three, 18 two, and 4 children only one medicine. Three patients were followed conservatively, all had grade 3 HSPN pathology, two had an outcome of Meadow’s grade A and one of B. Mycophenolate mofetil (MMF) was the most commonly used immunosuppressive agent other than steroids with a median duration of 9 (6, 16) months. Cyclophosphamide was used in six children, three times as one dose, twice as four, and once in five doses. Three patients were treated with azathioprine and four with a calcineurin inhibitor. Rituximab was trialed in two children. Drug side effects were observed in three patients receiving angiotensin converting enzyme inhibitor (ACE-I; hypotension, cough), and in one patient on MMF (mild neutropenia).

There were 20 patients whose outcome was classified as Meadow’s grade A, 24 as B, three as C, and two as D. During follow-up, no patient death was observed. The relationship between clinical and laboratory findings at onset and clinical outcome according to Meadow’s criteria is given in Table 3. Favorable outcome was significantly associated with early treatment initiation of both angiotensin converting enzyme inhibitors (ACE-I)/angiotensin receptor blockers (ARB) and steroids. Among patients with grade D outcome: one presented as NS, received oral steroids plus MMF, and had developed stage 5 CKD by the end of 24 months; another patient presented as NS-NephrS with an eGFR of 31 ml/min/1.73 m^2^, received steroids, cyclophosphamide, plasmapheresis without benefit and at 16th month kidney transplantation was performed. Initial kidney biopsies of both children showed grade 3 pathology. Among four patients whose biopsies showed grade 5 pathology, three had Meadow’s grade A outcome and one grade B. Using the Oxford classification, a statistically significant worse outcome was observed in children with segmental glomerulosclerosis (S score) (Table 3). In the favorable outcome group, there were five (25%) patients with eGFR below 90 ml/min/1.73 m^2^ and six (30%) patients with NS presentation, whereas in the unfavorable group (Meadow’s B–D), there were four (13.7%) patients with low GFR and three (10.3%) with NS presentation.

**Table 3:** Patient characteristics according to Meadow’s criteria

Meadow’s Score	Favorable outcome (A) *n* = 20	Unfavorable outcome (B-D) *n* = 29	*P* value
Gender, female male	8 (40%) 12 (60%)	16 (55.2%) 13 (44.8%)	0.296
Age at biopsy, years	8.6 (5)	10.5 (5.5)	0.122
Ethnicity			0.217
European descent	11 (55%)	19 (65.5%)	
Other ethnic groups	2 (10%)	4 (13.8%)	
Unknown (not asked/ not specified)	7 (35%)	6 (20.6%)	
eGFR at biopsy, ml/min/1.73 m^2^	146 (100.25)	138 (58.5)	0.823
Duration from HSPN to biopsy, months	0 (1.75)	2 (7.5)	0.001
Duration from HSP to HSPN, months	0.5 (1)	1 (1)	0.726
Duration from HSPN to ACE-I/ ARB treatment, months	0 (6)	4 (13.5)	0.018
Duration from HSPN to steroid treatment, months	0 (0)	1 (4.5)	0.011
Urine albumin/creatinine at biopsy, mg/mmol	553.5 (583)	236 (596)	0.070
Serum albumin at biopsy, g/L	30 (9)	31.5 (11)	0.160
Biopsy grade (ISKDC)			0.397
1	0 (0%)	1 (3.4%)	
2	2 (10%)	5 (17.2%)	
3	14 (70%)	18 (62.1%)	
4	1 (5%)	4 (13.8%)	
5	3 (15%)	1 (3.4%)	
Biopsy grade (Oxford classification)			
M1	19 (95%)	26 (89.7%)	0.502
E1	13 (65%)	15 (51.7%)	0.356
S1	3 (15%)	14 (48.3%)	0.031
T1	0 (0%)	0 (0%)	
C1	10 (50%)	14 (48.3%)	0.574
C2	8 (40%)	9 (31%)	
Clinical presentation			0.415
Mild proteinuria	1 (5%)	2 (6.9%)	
Nephrotic range proteinuria	3 (15%)	8 (27.6%)	
Nephrotic syndrome	6 (30%)	3 (10.3%)	
Nephritic syndrome	1 (5%)	1 (3.4%)	
Nephritic syndrome+nephrotic range proteinuria	1 (5%)	5 (17.2%)	
Nephritic+nephrotic syndrome	8 (40%)	10 (34.5%)	
ACE-I/ARBs	15 (75%)	26 (89.7%)	0.173
Pulse+oral steroids	10 (50%)	7 (24.1%)	0.062
Oral steroids	4 (20%)	16 (55.2%)	0.019
Steroids any	14 (70%)	23 (79.3%)	0.456
Immunosuppresive therapy other than steroids			0.632
- None	7 (35%)	15 (51.7%)	
- Mycophenolate mofetil	7 (35%)	8 (27.6%)	
- Other	4 (20%)	3 (10.3%)	
- Mycophenolate mofetil+other	2 (10%)	3 (10.3%)	

*ACE-I*, Angiotensin converting enzyme inhibitor; *ARB*, angiotensin receptor blocker; *eGFR*, estimated glomerular filtration rate; *HSPN*, Henoch-Schönlein Purpura Nephritis. Continuous data are presented as median (IQR: interquartile range); categorical data are given as frequency (percentage). Meadow’s grade A: normal, B: minor urinary abnormalities, C: active kidney disease, D: kidney insufficiency. Other immunosuppresive therapy: any of azathioprine, cyclophosphamide, cyclosporine, tacrolimus or rituximab

**Fig. 1:**
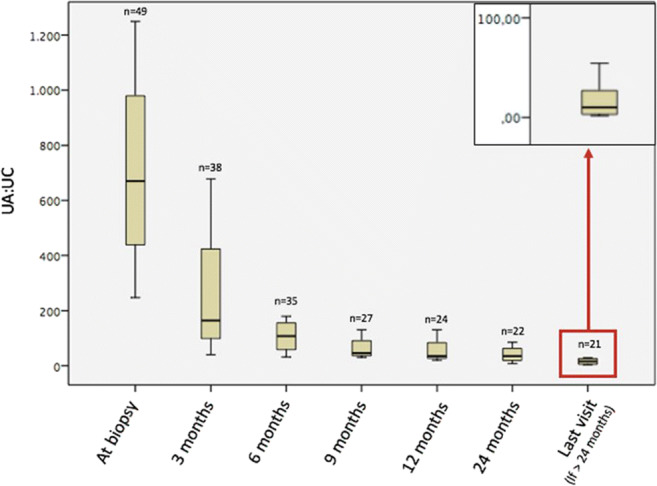
Urine albumin/creatinine ratios (median and IQR) vs. time

**Fig. 2:**
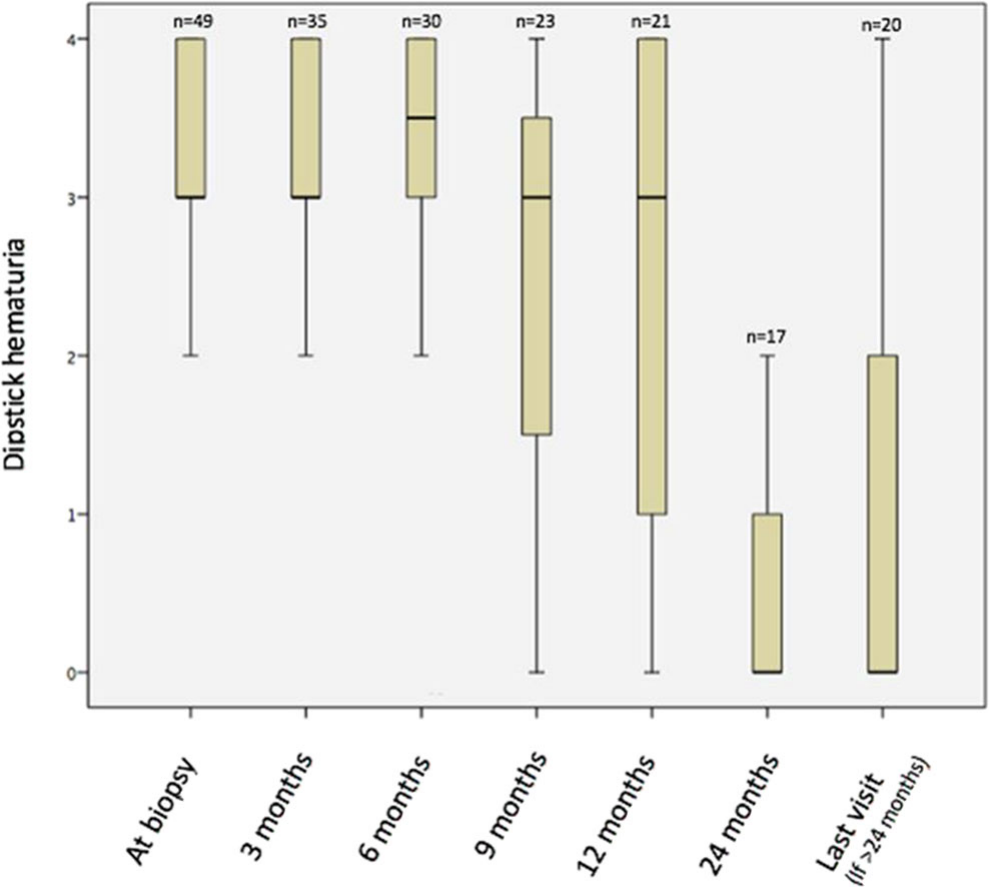
Dipstick hematuria (median and IQR) vs. time

Important clinical and laboratory characteristics of patients at last follow-up are given in Table 4. At that time, 48 children were normotensive, and only one had stage 1 HT, which was a significant improvement compared with 37 (75.5%) children with normal BP, eight (16.3%) with stage 1 and four (8.2%) with stage 2 HT at the time of biopsy (*p* = 0.004). Twenty-eight (57.1%) patients were free of albuminuria by 24 months and 34 patients at last visit (69.3%). Hematuria was cleared in 18 (36.7%) patients by 24 months and in 20 (40.8%) at last visit. The courses of hematuria and albuminuria during follow-up are depicted in Figs. 1 and 2.

**Table 4:** Clinical and laboratory characteristics of patients at last follow-up

Blood pressure	
Normal Stage 1 hypertension	48 (98%) 1 (2%)
eGFR, ml/min/1.73 m^2^	163.5 (44.0)
Urine albumin/creatinine, mg/mmol	18.5 (43.95)
Kidney function	
Normal	20 (41%)
Stage 1 CKD	24 (49%)
Stage 2 CKD	3 (6%)
Kidney failure	2 (4%)

CKD, Chronic kidney disease; eGFR, Estimated glomerular filtration rate. Continuous data are presented as median (IQR: interquartile range); categorical data are given as frequency (percentage)

## Discussion

In this study, we found that children with severe pathologies on kidney biopsy more often presented with nephrotic range proteinuria and hypoalbuminemia. Time to kidney biopsy from onset of HSPN and onset of treatment with ACEi and steroids were the only factors associated with a favorable outcome. Neither clinical presentation, nor choice of immunosuppressive treatment nor underlying pathology was linked to the final outcome. The rate of kidney failure was less than 5%.

The median duration from onset of HSP to onset of HSPN was 1 month in our present study, which is in accordance with the literature; 85% HSP kidney involvement within 4 weeks, 91% in 6 weeks, 97% within 6 months [9]. Severe HSPN may present with quite different clinical pictures. It is known that nearly all HSPN patients (> 95%) have hematuria at presentation and isolated hematuria has been reported in between 14 and 88% of cases [6, 25, 26]. Hematuria of various degrees were seen in all our patients (40/40). Nephrotic presentation in HSPN has been reported in between 15.9 and 21% of cases [25, 27]. In our study, NS was seen in 18.4% of the patients. A recent study showed that 19% of HSPN patients had NS at the time of biopsy, 32% nephrotic range proteinuria and 48% significant proteinuria, however the degree of proteinuria did not seem to effect the outcome [28]. Decreased creatinine clearance at HSPN presentation has been reported in between 1.4 and 45% of patients [25–27]. We found that 22.4% of the patients had an eGFR below 90 ml/min/1.73 m^2^ at presentation.

In the present study, 40% of patients were in complete remission at last follow-up, more than half had some degree of CKD with a low rate for kidney failure, less than 5%. At last visit, albuminuria had resolved in 70% of patients and hematuria in 40%. Different case series give varying results regarding progression to CKD [13, 29, 30]. Mir et al. [25] reported that the majority of patients had complete recovery at long-term follow-up and only 1.2% had developed kidney failure. Delbet et al. [28] reported that 70% of the children were in remission at the end of a median follow-up of 37 months and one-third ended up with some degree of kidney damage. Halling et al. [30] reported similar findings, with 26% having persistent kidney disease after a mean 5.2-year follow-up. The very variable outcome of HSPN might be due to many factors, such as different pathogenic pathways and patient-specific responses to treatments.

There are conflicting results on the relationship between the clinical severity and outcome. A systematic review reported that risk of long-term kidney impairment ranged between 0.5–3.8% for isolated hematuria and/or proteinuria, while this ratio increased to 11.1–31.7% for NephrS or NS [9]. Ronkainen et al. [31], with a mean follow-up of 24.1 years, reported that severe kidney presentation had poorer outcome. Similarly another study indicated that patients with favorable prognosis had higher eGFR levels at the time of biopsy [28]. There are however studies indicating that even mild kidney symptoms at onset could indicate a poor kidney prognosis [11, 32]. Our present study failed to show any predictive value for outcome of any clinical or laboratory findings at presentation. Although initial proteinuria, in our study, was more frequent in patients with severe pathology, it did not show a significant relationship to kidney prognosis. Similarly, Butani et al. [13] reported no features of presentation that were related to the kidney outcome.

Early kidney biopsy and treatment in pediatric HSPN has been suggested to be beneficial [27, 33]. Lesions seen on the kidney biopsies depend on the timing of the biopsy in relation to the onset of HSPN. Biopsies performed 30 days after the onset of HSPN were associated with more chronic lesions and lower eGFR [27]. Our present study also showed a significant association between early biopsy and favorable outcome. Interestingly, kidney outcome was however not affected by the severity of the underlying pathology graded according to ISKDC. Similar findings have been seen in other studies using the ISKDC classification, in which histological findings in the initial kidney biopsies could not predict kidney outcome [32, 34]. In contrast, Çakıcı et al. [35] found that kidney dysfunction at last follow-up was more commonly observed in patients with severe ISDKC pathology. Signs of chronic kidney changes as scored in the Oxford classification were however, both in our study and in the others, significantly associated with the outcome. These may be tubular atrophy and interstitial fibrosis [35] or glomerulosclerosis as in our study. The advantage with the Oxford classification is that it, in contrast to ISKDC, accounts not only for acute lesions but also chronic changes and thus shows the degree of irreversible damage. Jimenez et al. [36] aimed to evaluate the usefulness of MEST-C score in pediatric HSPN and were also able to show that the S score was significantly associated with worse kidney outcome. Our results were also in accordance with previous studies reporting that acute and generally transient lesions like M1, E1, and C1 were not significantly associated with kidney outcome [35, 37].

Consensus on HSPN treatment is still lacking and there are currently no widely accepted recommendations for treatment of moderately severe and severe HSPN in children. The KDIGO guideline for HSPN suggests that children with persistent proteinuria (> 0.5–1 g/day/1.73 m^2^) should be treated with ACE-I or ARB, and after a trial of ACE-I or ARB children with persistent proteinuria (> 1 g/day/1.73 m^2^) and GFR of > 50 ml/min/1.73 m^2^ may be given a 6 month-course of steroids. Pulse steroids, cyclophosphamide, and plasmapheresis may be considered in crescentic HSPN, NS, and/or impaired kidney function [38]. Some other studies also suggest MMF, azathioprine or cyclosporine A for HSPN treatment [29, 39, 40]. Reviews by leading experts have concluded that scientifically proven treatment does not exist for HSPN [41, 42]. One recent review gave 19 different recommendations on treatment of HSP [43]—all but one which showed a level of evidence of 4a and strength of recommendation of D meaning that all recommendations were based on expert opinion.

Tudorache et al. [32] also, like our study, reported that early initiation of ACE-I/ARB treatment improved the long-term outcome regardless of the initial histology. Similarly, we showed that early introduction of treatment, both ACE-I/ARB and steroids, was associated with improved final outcome. Treatment with ACE-I is recommended by all authors. In our study, a large proportion of children received steroids and ACE-I, however the kidney outcomes were comparable with those who did not receive that treatment. In our study, we could not show significant association between any treatment and the kidney outcome. This shows the difficulty of basing treatment recommendations on expert opinion, as it is difficult to get a large unequivocal experience on treatment of HSPN even in a large center like ours.

The main limitations of our study are a relatively small sample size and the retrospective nature of the analyses. Another limitation is the nonstandardized treatment used and relatively short follow-up period.

In conclusion, even in a large center, there are very low numbers of children developing kidney failure due to HSPN in the short to medium term. There are however a number children with CKD of differing severity who later might go on to develop kidney failure. In this study, no clinical or laboratory parameters could be shown to predict the long-term prognosis, nor could any superior treatment modality be defined. Larger scale studies—preferably prospective—are needed to define which children need to be treated, and which treatments are effective.
